# Automated titrations: the use of automated multiple flow injection analysis for the titration of discrete samples

**DOI:** 10.1155/S1463924681000084

**Published:** 1981

**Authors:** Kent K. Stewart, A. Gregory Rosenfeld

**Affiliations:** Nutrient Composition Laboratory Nutrition Institute, Human Nutrition Science and Education Administration U.S. Department of Agriculture Beltsville Maryland 20705 USA


### Automated titrations: the use of automated multiple flow injection analysis for the titration of discrete samples*

**Kent K. Stewart & A. Gregory Rosenfeld**
*Nutrient Composition Laboratory, Nutrition Institute, Human Nutrition, Science and Education Administration, U.S. Department of Agriculture, Beltsville, Maryland 20705, USA.*

### Introduction

In 1974 Fleet and Ho [1] successfully demonstrated an automated continuous flow titration system using a reagent gradient. This system had a throughput of only ten samples per hour, but it was significant since it demonstrated the principles for titration of discrete samples in continuously flowing streams. Nagy et al. [2,3] have also demonstrated a similar approach. In 1977, Ruzicka et al. [4] described the principles and an apparatus for the automated titration of manually injected samples using a gradient of the injected samples. A system is now described for automated titrations of discrete samples using a strategy [5] which automates the handling of discrete samples for flow injection analysis and Ruzicka's gradient titration system.

* *A preliminary report of this project was presented by K. K. Stewart and A. G. Rosenfeld at the 30th Pittsburgh Conference on Analytical Chemistry and Applied Spectroscopy, March 5-9, 1979, Abstract #657.*

### Equipment

The apparatus for the automated multiple flow injection analysis (AMFIA) titration is shown disgrammatically in Figure 1. The reagent pump system was a depulsed positive displacement pump described previously [6]. The sample insertion valve system utilised a pneumatically actuated 6-way valve (Valco Instrument Co. Inc., Houston, TX USA). Sample

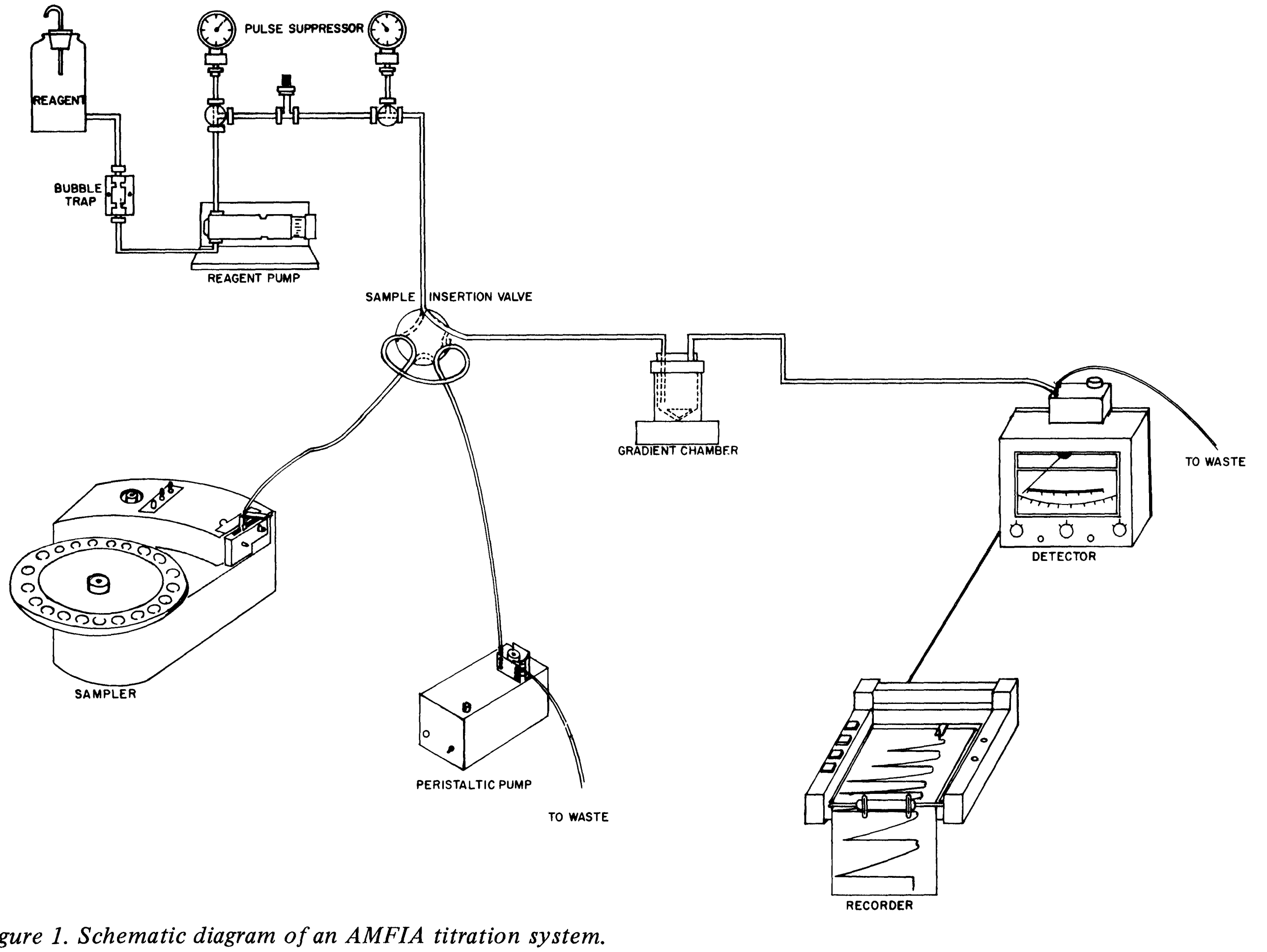

*Figure 1. Schematic diagram of an AMFIA titration system.*

loops were constructed from the appropriate lengths of 0.46mm i.d. Teflon tubing (Penntube Plastics Co., Clifton Heights. PA USA). Teflon tubing of 0.46 mm i.d. was used throughout the system, except in the peristaltic pump. The gradient chamber was easily made by a slight modification of a 500 μl reacti-Vial and an exterior thread chromatographic fitting (Wheaton Scientific, Millville, NJ USA). One hole was drilled at an angle into the side of the fitting to permit the insertion of the input tubing (0.46 mm i.d.). The inlet tubing was stretched, snugly fitted in the hole, and extended about 15 mm into the chamber. The titrant flow was in through the tubing and out through the chromatographic fitting. Similar gradient chambers have been constructed from 100 μl vials. The detector was constructed from an photometer (Aminco Scientific Co., Silver Spring, MD USA), a miniature end-lens lamp (Chicago Minature Lamp Works, Chicago, IL USA), 0-10 volt DC power supply (Sorenson Power Supply, Manchester, NH USA), and an 570 nm internal light filter (Dell Optics Co. Inc., North Bergen, NJ USA). A flow cell compartment was machined to fit into a photomultiplier tube housing, and to hold a liquid chromatographic flow cell (Schoeffel Instrument Corp., Westwood, NJ USA), the lamp, and the filter. The system response was electronically converted to absorbance in a manner similar to that described by Walker and Amandor [7].

Samples were aspirated from a sampler (Alkemp Corp., Clackamas, OR USA) into the sample insertion valve with a peristaltic pump. The pneumatic system was controlled with solenoid air valves (Laboratory Data Control, Riviera Beach, FL USA). Firing of the solenoid valves was synchronised with the movement of the sample probe. The appropriate solenoids were wired to the microswitch in the sampler. The inject solenoid was wired to the normally closed terminal and the common terminal of the microswitch, the load solenoid was wired to the normally open terminal and the common terminal of the microswitch. A full bridge rectifier (Motorola-MDA-920AA5) in each circuit provided the DC power needed for the solenoids. When the sample probe was moved to the wash reservoir, the sample tray was moved one step and the pneumatic system moved the sample insertion valve to the inject position; when the sample probe was moved into the sample cup, the sample insertion valve was moved to the load position.

The flow rate of the withdrawal pump was adjusted for each sampling rate so that the pick up bubble had cleared the sample loop and that during sampling the loop is full of solution. The pneumatic system was operated at approximately 100 psi and 1/8 in. diameter tubing and a ballast tank were used to minimise transit time of the sample valve.

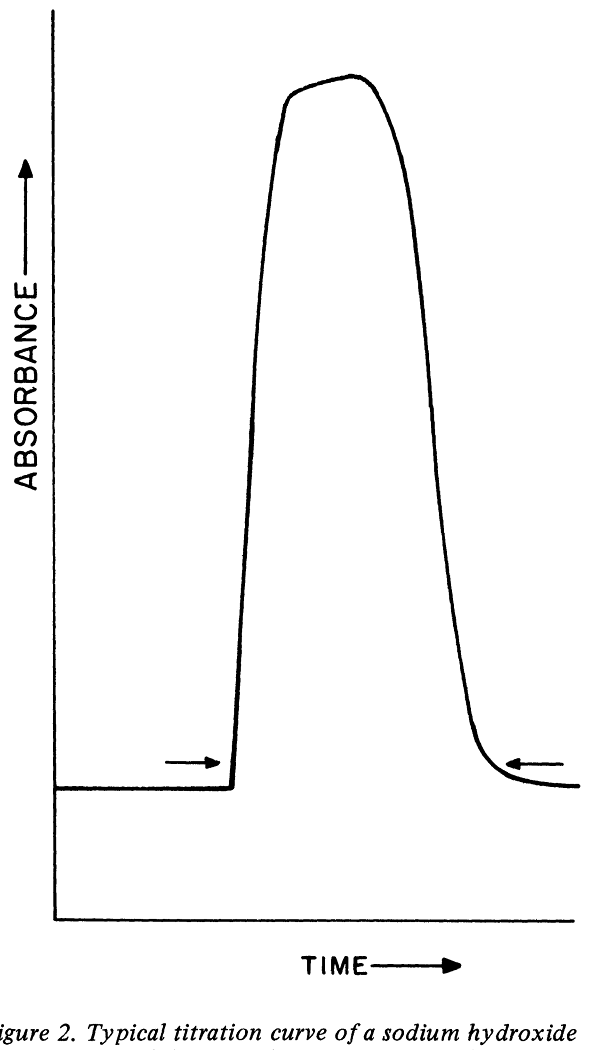

*Figure 2. Typical titration curve of a sodium hydroxide sample using sulphuric acid containing phenolphthalein as a titrant.*

### Results

The control of sample dispersion is critical to the development of the AMFIA-titration system. In a series of preliminary experiments, the three dispersion systems discussed by Ruzicka *et al* [4] were tested. That is, long tubes of small diameter, short tubes with large diameters and stirred gradient chambers. While each of these dispersion systems gave exponential dilutions under some conditions, it was found that the stirred gradient chambers were superior since these chambers exhibited exponential dilution behaviour over four orders of magnitude. The other systems rarely had exponential dilution behaviour over more than one order of magnitude.

To demonstrate the automation of the FIA titration system standard samples of sodium hydroxide solutions were titrated with solutions of sulphuric acid containing phenolphthalein. A typical sample curve is shown in Figure 2. The titration time was measured from just after the phenolphthalein pink colour appeared (first arrow) until just before it disappeared (second arrow). A typical standard curve is shown in Figure 3. These titrations were performed at 60 samples per hour. A linear plot of the logarithm of the sample concentration versus the peak width (in volume or time) is exactly equivalent to that reported by Ruzicka *et al.* [4] for the manual system. Likewise, investigations of the effect of flow rates on the standard curves yielded a series of straight lines with different slopes as reported previously [4].

The effect of the sample loop size was investigated and the results are shown in Figure 4. One significant point to note is that the standard curves were linear even when the sample loop volume (1 ml) was larger than the dilution chamber volume (0.5 ml).

### Discussion

The AMFIA-titration system is a simple, relatively inexpensive means of automating single end point titrations. The sample throughput of 60 samples per hour should be adequate for many analytical situation. The system compliments the manual system described by Ruzicka *et al.* especially since the two systems show similar responses with regard to the linearity of logarithm of concentration versus peak width, and with respect to the effect of flow rate.

Of the several factors that affect sample throughput, the most important are the size of the gradient chamber, flow rate of titrant, and ratio of titrant concentration to sample concentration. It has been found useful to use 2 to 10 μl sample loops, small gradient chambers (500 or 100 μl) and titrant flow rates of 5 to 10 ml per minute. These conditions allow the analyst flexibility to change the titrant concentration

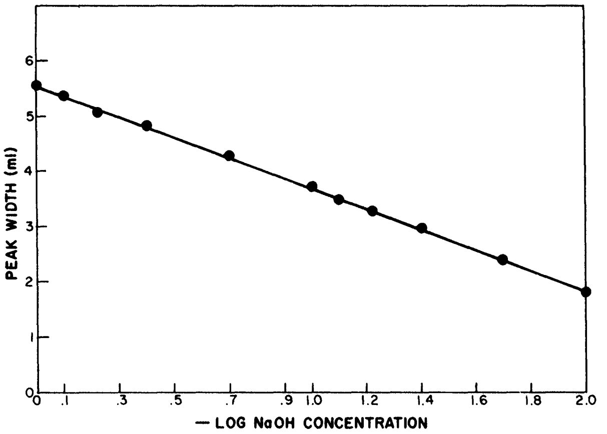

*Figure 3. Typical standard curve for AMFIA titration system. The titrant was 0.001N $H_2SO_4$ containing 0.002% phenolphthalein. The flow rate was 9.0 ml/minute and the sample rate was 60 samples per hour. The sample size was 200 µl and the dilution chamber had a volume of 500 µl.*

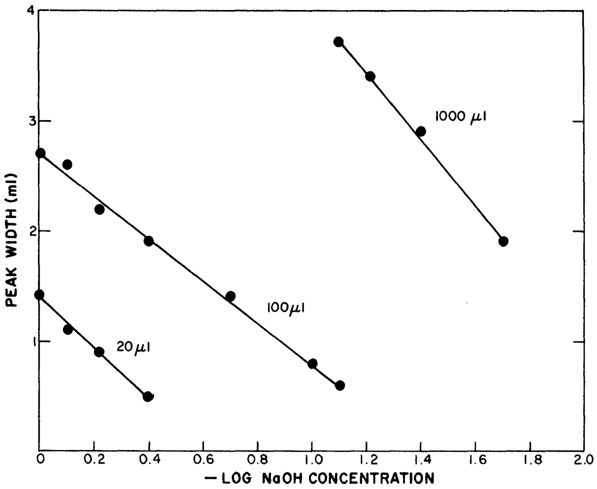

*Figure 4. Standard curves for AMFIA titration system with different sized sample loops. The titrant was 0.001N $H_2SO_4$ containing 0.002% phenolphthalein the flow rate was 8.0 ml/minute and the sampling rate was varied according to the sample loop volume. The dilution chamber had a volume of 500 µl. Each standard curve is labelled with the volume of the sample loop in micro-litres.*

and still permit a rapid sample throughput. For high accuracy and precision, samples of low concentration should be titrated with titrants of low concentration. The titrant concentrations were usually selected so that samples with the highest concentrations are titrated in less than 60 seconds. In this manner a throughput of 60 samples per hour can be maintained for the system when a fixed time schedule is used for sample pickup.

When samples in a single batch have widely varying concentrations, automatic feedback control is useful. With automatic feedback, a microprocessor system is used to control the sample insertion valve, the sample probe, the sample tray, and the data handling. The micro-processor monitors the detector signal. When the signal falls below a preset level, the next sample is aspirated into the sample insertion valve and inserted into the system. The automated feedback system permits the use of very dilute titrants, provides the capability of handling samples of greater concentration ranges and provides more accuracy and precision for the assay of dilute samples. A microprocessor control system that incorporates automatic feedback control has been described elsewhere [8].

This simple automated titration system could be useful in many different types of laboratories for large numbers of routine single end point titrations. In a series of studies to be reported later, it was found that many different detectors may be used. In most cases, the analyst may choose the detector that is appropriate for the chemical analyses and use it in the AMFIA system described here.

ACKNOWLEDGEMENTS
The authors gratefully acknowledge the knowledgeable technical assistance of Mrs. Darla Higgs and the skillful art work of Paul Padovano.

DISCLAIMER
*Mention of trademark of proprietary products does not constitute a guarantee or warranty of the product by the U.S. Department of Agriculture, and does not imply their approval to the exclusion of other products that may also be suitable.*

REFERENCES
[1] Fleet, B. and Ho, A.Y.W. *Analytical Chemistry,* 1974, **46**, 9-11.
[2] Nagy, G., Toth, K., and Pungor, E., *Analytical Chemistry,* 1975, **47**, 1460.
[3] Nagy, G., Feher, Z. Ast., Toth, K., and Pungor, E., *Analytica. Chemica Acta,* 1977, **91**, 87.
[4] Ruzicka, J., Hansen, E.H. and Mosback, H. *Analytica Chimica Acta,* 1977, **92**, 235-249.
[5] Stewart, K.K., Beecher, G.R. and Hare, P.E. U.S.Patent4,013,413, 1977.
[6] Stewart, K.K., *Analytical Chemistry,* 1977, **49**, 2125-2126.
[7] Walker, L. and Amador, E. *Clinica Chimica Acta,* **46**, 181-186, 1973.
[8] Stewart, K.K., Brown, J.F. and Golden, B.M. *Analytica. Chimica. Acta,* **114**, 1980, 119-127.

